# Experimental procedures to identify and validate specific mRNA targets of miRNAs

**DOI:** 10.17179/excli2015-319

**Published:** 2015-07-02

**Authors:** Terry S. Elton, Jack C. Yalowich

**Affiliations:** 1 College of Pharmacy, Division of Pharmacology, The Ohio State University, Columbus, OH, USA

**Keywords:** miRNAs, miRISC, miRNA/mRNA target prediction algorithms

## Abstract

Functionally matured microRNAs (miRNAs) are small single-stranded non-coding RNA molecules which are emerging as important post-transcriptional regulators of gene expression and consequently are central players in many physiological and pathological processes. Since the biological roles of individual miRNAs will be dictated by the mRNAs that they regulate, the identification and validation of miRNA/mRNA target interactions is critical for our understanding of the regulatory networks governing biological processes. We promulgate the combined use of prediction algorithms, the examination of curated databases of experimentally supported miRNA/mRNA interactions, manual sequence inspection of cataloged miRNA binding sites in specific target mRNAs, and review of the published literature as a reliable practice for identifying and prioritizing biologically important miRNA/mRNA target pairs. Once a preferred miRNA/mRNA target pair has been selected, we propose that the authenticity of a functional miRNA/mRNA target pair be validated by fulfilling four well-defined experimental criteria. This review summarizes our current knowledge of miRNA biology, miRNA/mRNA target prediction algorithms, validated miRNA/mRNA target data bases, and outlines several experimental methods by which miRNA/mRNA targets can be authenticated. In addition, a case study of human endoglin is presented as an example of the utilization of these methodologies.

## Introduction

MicroRNAs (miRNAs) are an endogenous family of single-stranded 20-25 nucleotide non-coding RNAs that play a critical role in posttranscriptional gene regulation by acting as guide molecules for the miRNA-induced silencing complex (miRISC) to inhibit gene expression by targeting specific mRNAs for translational inhibition and/or degradation (reviewed in Bartel, 2009[15]; Fabian et al., 2010[49]; Fabian and Sonenberg, 2012[48]; Ha and Kim, 2014[62]; Wilson and Doudna, 2013[167]). Newer regulatory roles for miRNAs have also been documented, including miRNA mediated induction of gene expression (reviewed in Valinezhad Orang et al., 2014[158]; Vasudevan, 2012[160]).

Currently, 2,588 mature miRNAs processed from 1,881 precursor miRNAs have been annotated in the human genome (miRBase: http://www.mirbase.org/index.shtml, release #21, June 14 2014). It is now clear that the expression levels of miRNAs vary widely; some are ubiquitously expressed, while others are expressed in a tissue- and/or cell-specific manner, and many show spatiotemporal expression patterns (Hausser et al., 2009[67]; Landgraf et al., 2007[90]; Moreau et al., 2013[117]). Importantly, individual miRNAs can act upon numerous target mRNAs and every mRNA can be targeted by multiple miRNAs; allowing for enormous combinatorial complexity and regulatory potential (Balaga et al., 2012[13]; Dombkowski et al., 2011[42]; Friedman et al., 2014[55]; Gurtan and Sharp, 2013[61]). Computational predictions suggest that more than 60 % of all human protein-coding mRNAs harbor at least one conserved miRNA-binding site, and many more mRNAs contain non-conserved miRNA binding positions, suggesting that the protein expression levels of the majority of human genes may be regulated by miRNAs (Friedman et al., 2009[54]). Therefore, it is not surprising that miRNAs play an integral role in almost all known biological processes (reviewed in Bartel, 2009[15]; Fabian et al., 2010[49]). Although miRNAs are thought to “fine-tune” gene expression since they generally repress protein levels less than 20 % (Baek et al., 2008[11]; Bartel, 2009[15]; Selbach et al., 2008[145]), recent studies suggest that miRNA function becomes more pronounced in response to physiologic and pathophysiologic stresses (reviewed in Leung and Sharp, 2010[96]; Mendell and Olson, 2012[114]).

## miRNA Biogenesis

**Figure 1:**
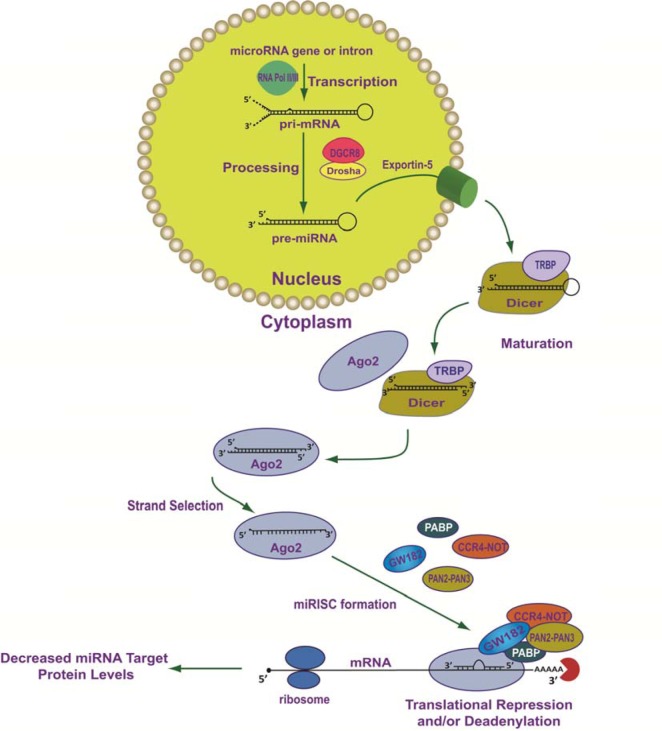
Mechanisms involved in miRNA biogenesis. This diagram includes miRNA transcription, maturation of miRNA/mRNA and two potential mechanisms for miRNA/mRNA silencing. The specific details describing these processes have recently been extensively reviewed (Fabian and Sonenberg, 2012; Ha and Kim, 2014; Krol et al., 2010; Wilson et al., 2013) and are briefly discussed in the text.

The vast majority of functional miRNAs are produced by a canonical multistep biogenic process which is initiated in the nucleus and is completed in the cytoplasm (Figure 1(Fig. 1); References in Figure 1: Fabian and Sonenberg, 2012[48]; Ha and Kim, 2014[62]; Krol et al., 2010[88]; Wilson et al., 2013[167]) (reviewed in Ha and Kim, 2014[62]; Krol et al., 2010[88]; Wilson and Doudna, 2013[167]). Mammalian miRNAs are embedded in primary miRNA transcripts (pri-miRNAs) which are 5′ 7-methylguanosine-capped (m7G-capped) and polyadenylated at their 3′ ends (Cai et al., 2004[28]; Lee et al., 2004[95]). Typically, pri-miRNAs are transcribed by RNA polymerase II from independent genes or from introns of protein-coding genes (Figure 1(Fig. 1)) (Cai et al., 2004[28]; Lee et al., 2004[95]). 

During the transcriptional process, pri-miRNAs fold into secondary configurations containing imperfectly base-paired stem-loops in which the mature miRNA sequences are embedded (Figure 1(Fig. 1)). Importantly, these hairpins serve as substrates for the Microprocessor complex (Figure 1(Fig. 1)). The Microprocessor complex is comprised of at least two proteins, Drosha (i.e., RNase III-type endonuclease) and its binding partner DGCR8 (DiGeorge syndrome critical region 8 gene) (Denli et al., 2004[36]; Gregory et al., 2004[58]; Han et al., 2004[65]; Lee et al., 2003[93]). DGCR8 is the Microprocessor component that directly interacts with pri-miRNAs through two double stranded RNA-binding domains (dsRBD) (Han et al., 2006[66]), while it's C-terminus interacts with Drosha (Yeom et al., 2006[171]). Drosha initiates the endonucleolytic cleavage of the stem-loop to release a “cropped” hairpin structured precursor miRNA (pre-miRNA) of ~60-70 nucleotides in length (Figure 1(Fig. 1)) (Lee et al., 2003[93]). Following Drosha/DGCR8 processing, the pre-miRNAs are bound by Exportin-5 (EXP5; encoded by the XPO5 gene) and a nuclear pore complex is formed with the GTP-binding nuclear protein, Ran-GTP (Figure 1(Fig. 1)) (Bohnsack et al., 2004[21]; Lund et al., 2004[103]; Yi et al., 2003[172]). Once the pre-miRNA is transported through the nuclear pore complex, GTP is hydrolyzed, the complex is disassembled, and the pre-miRNA is released into the cytoplasm. Subsequent to export from the nucleus, Dicer (another RNase III type enzyme) with its co-factor dsRBD protein, TRBP (TAR RNA-binding protein) (Chendrimada et al., 2005[31]) or PACT (protein activator of PKR) (Lee et al., 2006[94]), cleaves pre-miRNAs near the terminal loop resulting in miRNA duplexes of ~22 nucleotides (Figure 1(Fig. 1)) (reviewed in Ha and Kim, 2014[62]; Krol et al., 2010[88]; Wilson and Doudna, 2013[167]). The miRNA duplex is then released by Dicer and loaded onto Argonaute (AGO) protein which, together with the trinucleotide repeat containing 6A protein (TNRC6A/GW182), form the core of the miRISC (reviewed in Fabian and Sonenberg, 2012[48]; Ha and Kim, 2014[62]; Krol et al., 2010[88]; Wilson and Doudna, 2013[167]). 

During the AGO loading step, strand selection takes place and typically, the strand with the least thermodynamically stable base pair at its 5′ end in the miRNA duplex is selected as the “guide strand” (a single-stranded 20-25 nucleotide functional mature miRNA) and is retained within the miRISC (Figure 1(Fig. 1)) (Khvorova et al., 2003[83]; Schwarz et al., 2003[143]). In contrast, the “passenger strand” (also referred to as the complementary star-form miRNA strand or miRNA*) is released from miRISC and is subsequently degraded (Khvorova et al., 2003[83]; Schwarz et al., 2003[143]). It is important to note that passenger strands (miRNA*) are not always miRNA biogenic waste-products and they too can be loaded into miRISC, and exhibit inhibitory activity like any other miRNA (Chiang et al., 2010[33]; Okamura et al., 2008[125]; Packer et al., 2008[126]; Yang et al., 2011[170]). Given the increasing number of examples of “arm switching”, where two distinct functional mature miRNAs (guide strands) can be processed from opposite arms of the same pre-miRNA, these products are now denoted with the suffix -5p (from the 5′ arm) (e.g. miR-370-5p) or -3p (from the 3′ arm) (e.g. miR-370-3p) following their name. For more information regarding miRNA nomenclature as adopted by the miRNA Registry please see http://www.mirbase.org/help/nomenclature.shtml (Ambros et al., 2003[5]).

## miRNA/mRNA Silencing

After the mature miRNA is assembled into the miRISC, the guide strand targets this protein complex to specific mRNAs through a diffusion-controlled mechanism where the guide strand continuously binds/dissociates from mRNAs until a thermodynamically favorable binding site (i.e., miRNA recognition element, MRE) is found (Brown et al., 2005[27]). Importantly, this mechanism is facilitated when the guide strand has greater access to a given MRE harbored in a specific mRNA (Brown et al., 2005[27]). The association of miRNA with a specific mRNA target (i.e. miRNA:mRNA hybridization) involves a two-step process in which a miRNA anneals to a MRE and the mRNA structure then unfolds as the miRNA completes binding to a mRNA target (Long et al., 2007[101]).

With few exceptions, MREs are primarily located in the 3′-untranslated region (3′-UTR) of mRNAs and once recognized, mature miRNAs imperfectly base pair with MREs following a set of rules which have been experimentally and computationally identified (Brennecke et al., 2005[25]; Doench and Sharp, 2004[41]; Grimson et al., 2007[59]; Lewis et al., 2005[97]; Nielsen et al., 2007[122]). First, miRNA/mRNA target recognition involves Watson-Crick base pairing that must be perfect and contiguous at the 5′-end of the miRNA from nucleotides 2 to 7 and complementary to nucleotides in the 3′-UTR of mRNAs (Brennecke et al., 2005[25]; Doench and Sharp, 2004[41]; Lewis et al., 2005[97]). This zone represents the “seed” region and nucleates the miRNA-mRNA association. Second, a match to positions 2 to 7 of a miRNA (a 6mer seed match) usually has only a small effect on target mRNAs unless the seed sequence is flanked by a Watson-Crick match opposite position 8 (a 7mer-m8), an adenosine opposite position 1 (a 7mer-A1), or both (an 8mer) (Grimson et al., 2007[59]; Nielsen et al., 2007[122]). Third, the sequence context of the MREs in target mRNAs, also influence the functional importance of these sites (Grimson et al., 2007[59]; Nielsen et al., 2007[122]). For example, MREs harbored in the 5′-UTRs and/or open reading frames (ORFs) are much less effective as target sequences within these mRNAs for translational inhibition and/or degradation than those found in 3′-UTRs presumably because scanning or translating ribosomes interfere with miRISC binding (Grimson et al., 2007[59]). Additionally, miRNA efficacy can be improved if the MRE site is positioned within an AU-rich sequence region and not located in the middle of long unstructured 3′-UTRs probably reflecting areas in mRNAs less accessible to the miRISC (Grimson et al., 2007[59]; Kertesz et al., 2007[81]; Nielsen et al., 2007[122]). Finally, multiple MREs that are located within the same 3′-UTR tend to multiplicatively affect mRNA down-regulation via miRNA/mRNA binding (Grimson et al., 2007[59]). Specifically, when MREs are located within 8 to 40 nucleotides of each other, then miRNAs tend to act cooperatively, providing a potent increase in miRISC efficacy (Grimson et al., 2007[59]). Interestingly, MREs for a specific miRNA located in ORFs can also enhance regulation mediated by the same miRNA targeted MREs in 3′-UTRs (Fang and Rajewsky, 2011[51]).

After miRNA/mRNA target recognition, miRISC-bound target mRNAs are subjected to translational repression (i.e. inhibition of translation initiation) and/or deadenylation, decapping, and subsequent decay by a number of silencing factors that are scaffolded to this complex by TNRC6A/GW182 (Figure 1(Fig. 1)) (reviewed in Fabian and Sonenberg, 2012[48]). Importantly, global ribosome profiling studies which can precisely compare changes in translational efficiency to changes in mRNA levels, demonstrate that regardless of the miRNA, cell type, growth condition, or translational state, the majority of the negative post-transcriptional gene regulatory effects of miRNAs/miRISCs occur through mRNA decay (66 % - > 90 %) (Eichhorn et al., 2014[45]).

One major question regarding miRNA function that has not been adequately addressed is how miRNA concentration relates to target mRNA suppression. Several studies have suggested that only highly expressed miRNAs are able to mediate negative post-transcriptional gene regulatory effects (Brown et al., 2007[26]; Hafner et al., 2010[64]; Mullokandov et al., 2012[118]). For example, Mullokandov et al. (2012[118]) demonstrated that 60 % of the miRNAs detected by deep-sequencing had no discernible suppressive activity using a Sensor-seq assay which supported their earlier observation that miRNAs expressed below ~100 copies per cell had little regulatory capacity (Brown et al., 2007[26]). Finally, competitive endogenous RNAs (ceRNAs), which include transcripts with multiple MREs such as pseudogenes, long noncoding RNAs (lncRNAs), and miRNAs are now considered to be natural decoys or sponges which compete for common miRNAs and can therefore influence the expression levels of multiple miRNAs (reviewed in Salmena et al., 2011[140]). It is now hypothesized that miRNAs and ceRNAs can regulate each other in extended cross-talk networks and may play a major role in complex physiological processes and disease states (Salmena et al., 2011[140]). 

## miRNA/mRNA Target Prediction Algorithms

Given the large number of potential MREs harbored within any mRNA, computational miRNA/mRNA target algorithms are the most practical and efficient methods for identifying putative miRNA/mRNA interactions and selecting potential target sites for hypothesis conception and subsequent experimental validation (Alexiou et al., 2009[4]). There are a wide variety of prediction algorithms available to investigators that encompass a range of different computational approaches; however, the main prediction features include experimentally determined miRNA/mRNA pairing criteria necessary for optimal silencing (see above section). These include seed sequence match (Brennecke et al., 2005[25]; Grimson et al., 2007[59]; Krek et al., 2005[87]; Lewis et al., 2003[98], 2005[97]; Nielsen et al., 2007[122]), seed sequence conservation (Lewis et al., 2003[98]), target site accessibility (Mahen et al., 2010[106]), target site context (Grimson et al., 2007[59]; Kertesz et al., 2007[81]; Nielsen et al., 2007[122]), and free energy calculations (Yue et al., 2009[175]). Recently, prediction algorithms have also begun to implement machine learning approaches utilizing training data sets based on experimental information that represent positive and negative interactions to develop models of miRNA/mRNA targeting (Chi et al., 2009[32]; Hafner et al., 2010[64]). These models are subsequently utilized as part of the miRNA/mRNA target prediction process (Vlachos and Hatzigeorgiou, 2013[163]). Due to the differences in the computational approaches described above, the predicted miRNA/mRNA targets vary widely depending upon which algorithm is employed. How, therefore, can a research investigator determine which algorithm(s) should be utilized to identify potential miRNA/mRNA targets? 

To begin to address this critical question, several recent review articles have compared and contrasted many of the miRNA/mRNA target algorithms currently available (Alexiou et al., 2009[4]; Dweep et al., 2013[43]; Peterson et al., 2014[131]; Reyes-Herrera and Ficarra, 2012[137]; Vlachos and Hatzigeorgiou, 2013[163]). For example, in their original publication, Alexiou et al. (2009[4]) compared eight of the most commonly used human and mouse miRNA/mRNA target algorithms and suggested that, based on precision and sensitivity levels, that the top four algorithms were; DIANAmicroT 3.0 (http://microrna.gr/microT) (Maragkakis et al., 2009[108]), TargetScan (http://www.targetscan.org) (Friedman et al., 2009[54]), Pictar (http://pictar.org) (Lall et al., 2006[89]), and ElMMo (http://www.mirz.unibas.ch) (Gaidatzis et al., 2007[56]). The latest review article from this laboratory (Vlachos and Hatzigeorgiou, 2013[163]) provided a detailed overview of the major miRNA/mRNA target computational approaches utilized by TargetScan (http://www.targetscan.org, release 6.2, based on miRBase Release 17) (Garcia et al., 2011[57]; Grimson et al., 2007[59]), DIANA-microT-CDS (http://www.microrna.gr/microT-CDS, 5^th^ version, based on miRBase Release 18) (Paraskevopoulou et al., 2013[128]; Reczko et al., 2012[136]), and the miRanda-mirSVR algorithm (http://microrna.org, a database of predicted miRNA/mRNA targets based on miRBase Release 15) (Betel et al., 2010[17]). Additionally, Peterson et al. (2014[131]) recently concluded that, based on ease of use, range of capabilities, and relatively current input data, and maintenance of the software, the utilization of DIANA-microT-CDS, miRanda-mirSVR, or TargetScan was preferred for miRNA/mRNA target predictions. Given that only Diana-microT-CDS (Reczko et al., 2012[136]) and TargetScan (Garcia et al., 2011[57]; Grimson et al., 2007[59]) have been updated and significantly modified in the past several years, we advocate the use of these two algorithms to predict human and mouse miRNA/mRNA targets. Due to the rapid discovery rate of novel miRNAs (2,588 annotated human mature miRNAs, miRBase Release 21, http://www.mirbase.org) even these two algorithms would benefit from more current data input. 

It is also significant to note that updated algorithms identify up to 60 % of all available miRNA/mRNA targets and provide only one valid target in approximately every three predicted targets (Vlachos and Hatzigeorgiou, 2013[163]). It is evident, therefore, that even the best available algorithms still fail to identify a significant number of biologically important miRNA/mRNA targets (Reczko et al., 2012[136]). For example, several recent studies have demonstrated that non-canonical miRNA interactions are diverse, functional, much more prevalent than previously appreciated, and cannot be identified by any current algorithm (Grosswendt et al., 2014[60]; Helwak et al., 2013[68]; Martin et al., 2014[110]; Tan et al., 2014[153]). 

## miRNA/mRNA Target Prediction Algorithm Analyses: Endoglin as an Example

**Figure 2:**
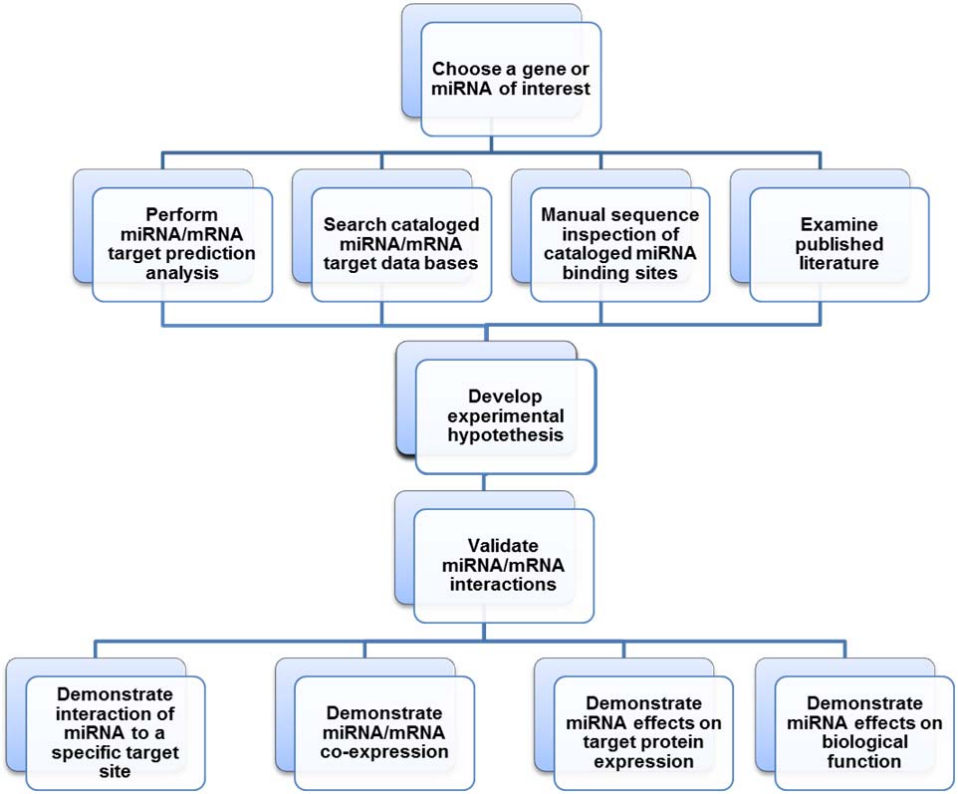
Proposed flow diagram for the identification of putative microRNA/mRNA target interactions, and subsequent hypothesis driven experimental validation of these predicted MREs.

Given the above review of miRNA biology and miRNA/mRNA target prediction algorithms, we propose the following “work flow” scheme (Figure 2(Fig. 2)) for the identification and validation of miRNA/mRNA target interactions. First, the investigator must choose a gene target or miRNA of interest to investigate.

For a case study we have chosen to analyze the human endoglin gene (ENG) for potential MREs. Endoglin (CD 105, TGF-β receptor III) is a homodimeric co-receptor for transforming growth factor beta (TGFβ) and is known to play a regulatory role in TGFβ signaling (reviewed in Kapur et al., 2013[78]; López-Novoa and Bernabeu, 2010[102]; Nachtigal et al., 2012[119]; Rosen et al., 2014[139]). It has been demonstrated that endoglin plays a role in many pathological processes, including cancer, angiogenesis, hereditary hemorrhagic telangiectasia (HHT), pre-eclampsia, pulmonary hypertension, heart failure, myocardial infarction, atrial fibrillation, atherosclerosis, and diabetes mellitus (Kapur et al., 2013[78]; Lee et al., 2012[92]; López-Novoa and Bernabeu, 2010[102]; Nachtigal et al., 2012[119]; Rosen et al., 2014[139]). Given that miRNAs play an integral role in most physiologic and pathophysiologic conditions (reviewed in Acunzo et al., 2015[1]; Adams et al., 2014[2]; Arunachalam et al., 2015[8]; Neppl and Wang, 2014[120]), it is of interest to determine whether or not endoglin expression is aberrantly regulated by miRNAs in certain disease states.

Once the gene target (in this case, endoglin) or miRNA of interest has been chosen, it must then be analyzed by miRNA/mRNA target prediction algorithms (Figure 2(Fig. 2)). Both the Diana-microT-CDS (Paraskevopoulou et al., 2013[128]; Reczko et al., 2012[136]) and TargetScan (Garcia et al., 2011[57]; Grimson et al., 2007[59]) algorithms allow the investigator to enter a specific ''Gene Symbol'' or “miRNA”. The predicted MREs harbored within the mRNA or all of the mRNAs which harbor a given MRE will then be computed. 

The Diana-microT-CDS algorithm results include; the identified miRNAs and their predicted location of MREs both in the coding sequences (CDS) and in the 3′-UTR, the seed sequence binding type, whether or not the predicted MRE is conserved (MREs that are conserved during evolution tend to be more biologically consequential compared to those that haven't, Friedman et al., 2009[54]), what species harbor the conserved MRE, the miTG (miRNA targeted genes) targeting score (the higher the score the higher the probability of targeting MREs harbored within the CDS or in the 3′-UTR of the human S-endoglin mRNA), and whether or not a given miRNA target is also predicted by the miRanda or TargetScan algorithms (Paraskevopoulou et al., 2013[128]). It is essential to note that this algorithm (Diana-microT-CDS) only analyzes the longest annotated transcript (i.e. the one with the longest 3′-UTR sequence) for each gene (Ensembl version 69, www.ensembl.org) (Paraskevopoulou et al., 2013[128]). This is a crucial consideration given that more than 90 % of human genes are estimated to undergo alternative splicing (Pan et al., 2008[127]; Wang et al., 2008[166]) and ∼70 % of all human genes contain multiple alternative cleavage and polyadenylation sites (Derti et al., 2012[37]). Importantly, these post-transcriptional regulatory mechanisms can result in mRNA isoforms that differ in CDS and/or in 3′-UTR length, and as a consequence, mRNA/miRNA target interaction sites can be added or subtracted from each isoform (Boutet et al., 2012[22]; Park et al., 2011[129]; Sandberg et al., 2008[141]; Tan et al., 2007[152]). Therefore, it is recommended that one has a clear understanding whether the gene of interest is regulated by alternative RNA processing mechanisms before performing miRNA algorithm analyses.

Interestingly, the human ENG gene generates two distinct mRNAs through alternative splicing, which results in isoforms that differ in a portion of their CDS and 3′-UTR (Bellón et al., 1993[16]; Pérez-Gómez et al., 2005[130]). The predominant human endoglin mRNA isoform is comprised of 15 exons, encodes a protein of 658 amino acids which has a cytoplasmic domain of 47 residues (long, or L-endoglin), and harbors a 670 nucleotide (nt) 3′-UTR (Bellon et al., 1993[16]). In contrast, the second human endoglin mRNA isoform is comprised of the same 15 exons, however, intron 14 is retained (Bellon et al., 1993[16]). The retention of intron 14 introduces a premature stop codon in the reading frame, therefore, this isoform encodes a protein of 625 amino acids which contains a cytoplasmic tail of only 14 residues (short or S-endoglin) and harbors a 905 nt 3′-UTR (Bellon et al., 1993[16]). The initial 235 nts of the 3′-UTR are unique to this isoform and the remaining 670 nucleotides overlap with the entire 3′-UTR of L-endoglin mRNA isoform (Bellon et al., 1993[16]). Although both endoglin forms are able to bind ligand (Bellon et al., 1993[16]), it is assumed that the structural differences of their cytoplasmic domains account for the distinct functional effects observed for each isoform (Aristorena et al., 2014[7]; Blanco et al., 2005[20], 2008[18], 2015[19]; Velasco et al., 2008[161]). 

Since the mRNA isoform which encodes S-endoglin harbors the longest 3′-UTR, the Diana-microT-CDS algorithm will only utilize this sequence for computing miRNA/endoglin mRNA target interactions. When this analysis is performed, a total of 259 (threshold set to 0.4) miRNAs are predicted to interact with the human S-endoglin mRNA isoform at 797 individual MREs, with 186 target sites located in the CDS and 611 sites in the 3′-UTR (data not shown). Table 1(Tab. 1) lists the top fifteen Diana-microT-CDS predicted miRNAs to interact with this mRNA isoform, and includes the number of predicted MREs and locations, and their respective miTG scores. Although 596 of the 611 predicted human S-endoglin MREs harbored in the 3′-UTR are conserved across at least one other species, this does not always mean that predicted target interactions are conserved between humans and lower species such as rodents. This is a key consideration given that *in vivo* miRNA/mRNA target validation experiments can't be performed if these MREs are not conserved in mice (see experimental validation sections below). As a result, Table 1(Tab. 1) includes whether any of the top fifteen identified miRNAs with predicted MREs harbored in the human S-endoglin 3′-UTR are also conserved in the mouse Eng gene. Interestingly, of the 259 Diana-microT-CDS identified miRNAs only 87 bind to 98 predicted MREs that are conserved in both the human and mouse ENG/Eng gene. It is also significant to note, however, that conservation of a miRNA binding site harbored in a given mRNA target is not a requirement for a functional miRNA (Witkos et al., 2011[168]). 

In contrast to Diana-microT-CDS, the TargetScan algorithm results include the identified miRNAs and the predicted location of MREs in the 3′-UTR but not in the CDS. However, this tool does allow the user to analyze any annotated splice variant for a given gene. For example, TargetScan will analyze both L-endoglin and S-endoglin 3′-UTRs. The TargetScan results also include the number and type of seed match of conserved and poorly conserved miRNA binding sites, and a total context score (predicted efficacy of targeting) (Garcia et al., 2011[57]; Grimson et al., 2007[59]). When the human S-endoglin mRNA isoform (NM_000118, 905 nt 3′-UTR) is analyzed by TargetScan, 152 miRNAs and/or miRNA families are predicted to interact with 189 MREs in the 3′-UTR. Table 2(Tab. 2) lists the top fifteen TargetScan predicted miRNAs to interact with this mRNA isoform, and includes the number of predicted MREs, the number of highly conserved MREs, the number of conserved mouse S-endoglin MREs, and their respective context scores. When the human L-endoglin mRNA isoform (NM_001114753, 670 nt 3′-UTR) is analyzed by TargetScan, 132 miRNAs and/or miRNA families are predicted to interact with 162 MREs in the 3′-UTR. Table 3(Tab. 3) lists the top fifteen TargetScan predicted miRNAs to interact with this mRNA isoform and includes the same information as shown in Table 2(Tab. 2). The data shown in these two tables is very similar, however, due to differences in the lengths of the analyzed 3′-UTRs the “target site context” (Grimson et al., 2007[59]) differs and this leads to changes in their total context scores and therefore the order of the predicted miRNAs. 

Given that the initial 235 nts of the human S-endoglin mRNA 3′-UTR are unique to this isoform, Targetscan identified 20 miRNAs and/or miRNA families that are predicted to target 20 MREs located only in this region (Table 4(Tab. 4)) and an additional 7 miRNAs and/or miRNA families that are predicted to interact with MREs located in this unique 3′-UTR region and in the 3′-UTR region common to both human endoglin mRNA isoforms (Table 5(Tab. 5)). It is possible to utilize the lists shown in Tables 4(Tab. 4) and 5(Tab. 5) to devise an experimental hypothesis regarding potential miRNA regulatory differences between human S- and L-endoglin mRNAs.

As described earlier, one of the parameters computed by the Diana-microT-CDS algorithm is whether or not a given miRNA/mRNA target is also predicted by the TargetScan algorithm (Paraskevopoulou et al., 2013[128]). Interestingly, this algorithm found that only 18 of the predicted 259 miRNAs overlapped with miRNAs computed by TargetScan to interact with human endoglin mRNAs. However, by direct (manual) comparison of the miRNA/endoglin mRNA target site interactions computed by the Diana-microT-CDS and TargetScan algorithms, over 50 % of these sites overlapped (data not shown). Table 6(Tab. 6) lists the top 20 miRNAs and/or miRNA families predicted by both algorithms based on targeting scoring. Importantly, this list of miRNAs predicted by both algorithms to target human endoglin mRNAs can be utilized to begin to formulate experimental hypotheses.

## Analysis of Experimentally Supported miRNA/mRNA Target Data Bases: the Endoglin Example

Before one can develop “experimental hypotheses” to help guide research efforts regarding the validation of the specific miRNA/mRNA target interactions identified above, it is important to examine data sets of manually cataloged miRNA/mRNA interactions which are supported experimentally (Figure 2(Fig. 2)) (Hsu et al., 2014[70]; Sethupathy et al., 2006[146]; Vergoulis et al., 2012[162]; Vlachos et al., 2015[164]; Xiao et al., 2009[169]). The first available database of experimentally supported miRNA/mRNA targets was DIANA-TarBase (http://www.microrna.gr/tarbase) (Sethupathy et al., 2006[146]). DIANA-TarBase v7.0 was recently released and includes miRNA/mRNA interactions which have been manually curated from information fragmented and buried in thousands of published studies and raw next-generation sequencing (NGS) data sets on 356 different cell types from 24 species (Vlachos et al., 2015[164]). 

The curated data sets contain > 7,500 miRNA/mRNA interactions obtained from “low yield” experimental techniques (e.g. reporter genes, Northern Blotting, qPCR, Western Blotting, ELISA, and 5′ RLM-RACE) and > 500,000 interactions derived from high-throughput experiments (e.g. pSILAC, CLIP-Seq/CLASH, Degradome-Seq, AGO-IP, biotin pull-down, miTRAP, 3′ Life, and IMPACTSeq) (Vlachos et al., 2015[164]). At least two other similar databases, miRTarBase (http://mirtarbase.mbc.nctu.edu.tw/) (Hsu et al., 2014[70]) and miRecords (http://mirecords.umn.edu/miRecords/) (Xiao et al., 2009[169]), are also available. Notably however, these two databases have cataloged smaller sets of interactions, 51,460 and 2,705, respectively. Given that DIANA-TarBase v7.0 harbors significantly more entries than any other relevant database, we advocate for its use to survey experimentally supported miRNA/mRNA interactions.

To investigate the curated data which support miRNA/endoglin mRNA target interactions, the ''ENG” gene symbol was entered into the DIANA-TarBase v7.0 and all the cataloged interactions were identified. Table 7(Tab. 7) (References in Table 7: let-7b-5p: Selbach et al., 2008[145]; miR-16-5p: Balakrishnan et al., 2014[14]; miR-20a-3p: Balakrishnan et al., 2014[14]; miR-23b-5p: Balakrishnan et al., 2014[14]; miR-29a-5p: Balakrishnan et al., 2014[14]; miR-103a-3p: Balakrishnan et al., 2014[14]; miR-107: Balakrishnan et al., 2014[14]; miR-532-5p: Haecker et al., 2012[63]; miR-628-5p: Balakrishnan et al., 2014[14]; miR-522-3p: Tan et al., 2014[153]) documents ten human experimentally supported miRNA/endoglin mRNA interactions, and the methodology utilized to substantiate the interaction, the tissue and/or cell line used for experimentation, the location of the MRE if known, the type of interaction (direct or indirect), and the literature reference. Interestingly, three types of experimental methodologies were utilized to confirm miRNA/endoglin mRNA interactions: pSILAC, HITS-CLIP, and IMPACT-Seq (Balakrishnan et al., 2014[14]; Haecker et al., 2012[63]; Selbach et al., 2008[145]; Tan et al., 2014[153]) (Table 7(Tab. 7)). Briefly, pSILAC (pulsed stable isotope labelling with amino acids in cell culture) methodology involves the transfection of a given miRNA mimic into a cell line of choice that have been isotopically labeled or non-labeled and followed by mass-spectrometry-based proteomics to measure changes in protein production (Selbach et al., 2008[145]). pSILAC does not establish whether the reduction in a given protein results from the miRNA directly binding to a MRE harbored within an mRNA of interest (Selbach et al., 2008[145]). In contrast, the HITS-CLIP (high**-**throughput sequencing of RNA isolated by crosslinking immunoprecipitation) experimental approach involves the transfection of a given miRNA mimic into a cell line of choice followed by ultraviolet (UV) cross-linking to generate AGO/miRNA/RNA cross-linked regions (see Figure 1(Fig. 1)). The cross-linked RNAs are subsequently immunoprecipitated (IP) with AGO specific antibodies, the RNA is then extracted and subjected to high-throughput NGS (Balakrishnan et al., 2014[14]; Haecker et al., 2012[63]). Importantly, HITS-CLIP can identify direct miRNA/mRNA interactions. However, it is more difficult to pinpoint MRE locations located within the “pulled down” mRNA (Balakrishnan et al., 2014[14]; Haecker et al., 2012[63]) when compared to the IMPACT-Seq (identification of MREs by pull-down and alignment of captive transcripts-sequencing) methodology described below. The, IMPACT-Seq procedure involves the transfection of a given biotinylated miRNA mimic into a cell line of choice. MiRNA/mRNA targets are then pulled down utilizing streptavidin. The product is subsequently treated with RNase, and MREs are then identified by NGS of RNase-resistant fragments (Tan et al., 2014[153]). This experimental method not only results in the direct identification of miRNA/mRNA targets but also leads to the characterization of MRE(s) located within the targeted mRNA (Tan et al., 2014[153]). 

Cataloged results using pSILAC indicated that let-7b-5p decreased endoglin expression (Table 7(Tab. 7), Selbach et al., 2008[145]); yet neither Diana-microT-CDS nor TargetScan analysis of human endoglin mRNAs identified let-7b-5p MREs (not shown). These results suggest that let-7b-5p may not directly bind with specific MRE(s) harbored within this mRNA. Rather, this analysis supports the hypothesis that let-7b-5p indirectly decreases human endoglin expression. Therefore, to investigate whether endoglin expression is directly or indirectly regulated by this miRNA, the human S-endoglin mRNA isoform was manually screened for putative let-7b-5p MREs. Although let-7b-5p MREs were not located in the 3′-UTR, two putative CDS let-7b-5p MREs were identified 884 nts (5′ ACCUCA 3′, 6mer “seed” region) and 1361 nts (5′ CUACCUCA 3′, 8mer “seed” region) downstream from the start codon. It is not clear why the Diana-microT-CDS algorithm did not identify the putative let-7b-5p binding sites harbored in the CDS of this mRNA isoform. Hence, the complementary use of manual screening for putative MREs remains a viable strategy for hypothesis development and subsequent experimental designs. 

Nine miRNA/human endoglin mRNA target sites (Table 7(Tab. 7)) were identified by techniques (HITS-CLIP and IMPACT-Seq) that require the direct interaction of these miRNAs with the detected mRNA. Hence, specific MRE sequence(s) must be identifiable within endoglin mRNAs (Balakrishnan et al., 2014[14]; Haecker et al., 2012[63]). It was surprising, therefore, that only two out of nine miRNAs from Table 7(Tab. 7) (miR-16-5p and miR-628-5p) were predicted to interact with human endoglin mRNAs by the Diana-microT-CDS algorithm. This algorithm computed that the human S-endoglin mRNA isoform harbors three miR-16-5p MREs, one site was identified (5′ GCUGCU 3′, 6mer “seed” region) in the 3′-UTR, 842 nts downstream from the stop codon and two additional sites were identified in the CDS (5′ UGCUGCU 3′, 7mer “seed” region), 34 and 473 nts downstream from the start codon. Additionally, this mRNA isoform was predicted to have three miR-628-5p MREs, two sites were identified (5′ CAGCAU 3′, 6mer “seed” region) in the 3′-UTR, 184 and 223 nts downstream from the stop codon (within the intron 14 sequence) and one additional site was identified in the CDS (5′ UGUCAGCA 3′, 8mer “seed” region), 1645 nts downstream from the start codon. Interestingly, the human L-endoglin mRNA would not include the two miR-628-5p MREs harbored in the 3′-UTR because this mRNA isoform does not harbor intron 14. In contrast to results from the Diana-microT-CDS algorithm, TargetScan did not predict any of the ten miRNAs identified to interact with human endoglin mRNAs (Table 7(Tab. 7)), including the 3′-UTR miR-16-5p or miR-628-5p MRE. 

Although neither algorithm identified human endoglin mRNA MRE target sites for HITS-CLIP validated miRNAs (miR-20a-3p, miR-23b-5p, miR-29a-5p, miR-103-3p, miR-107, and miR-532-5p) (Table 7(Tab. 7)), human endoglin mRNAs were manually screened for these putative miRNA binding sites given the failure rate of prediction algorithms (Reczko et al., 2012[136]). When the human S-endoglin mRNA isoform was subjected to manual sequence analysis two putative miR-20a-3p CDS MREs (5′ AUGCAG 3′, 6mer “seed” region) were located (804 and 1239 nts downstream from the start codon) and two potential miR-23b-5p MREs (5′ AACCCA 3′, 6mer “seed” region) were identified in the 3′-UTR (448 and 509 nts downstream from the stop codon) of this mRNA isoform. Additionally, a miR-29a-5p MRE (5′ AAAUCAG 3′, 7mer “seed” region) was detected in the human S-endoglin mRNA 3′-UTR (882 nt downstream from the stop codon). It was also observed that two putative miR-532-5p CDS MREs (5′ AGGCAU 3′ and 5′ GGCAUG 3′, 6mer “seed” regions) located 247 and 1236 nts downstream from the start codon. Finally, sequence analysis detected three identical miR-103-3p and miR-107 MREs harbored in the human S-endoglin mRNA which overlaped with the three Diana-microT-CDS algorithm predicted miR-16-5p MREs above (3′-UTR MRE [5′ GCUGCU 3′, 6mer “seed” region] 842 nts downstream from the stop codon and two CDS MREs [5′ UGCUGCU 3′, 7mer “seed” region] 34 and 473 nts downstream from the start codon). It is now clear that miR-16-5p, miR-103-3p, and miR-107 belong to a group of paralogous, evolutionarily-conserved miRNAs termed the miR-15/107 family (Finnerty et al., 2010[53]). The miR-15/107 family includes miR-15a-5p, miR-15b-5p, miR-16-5p, miR-103-3p, miR-107 (which are expressed in all vertebrates), miR-195-5p, miR-424-5p, miR-497-5p, miR-503-5p (which are expressed in mammals), and miR-646 (human specific) (Finnerty et al., 2010[53]). Importantly this group of miRNAs shares a sequence (5′ AGCAGC 3′) near the 5′ end that complements with the Diana-microT-CDS algorithm predicted miR-16-5p MREs (5′ GCUGCU 3′) and the manually identified miR-103-3p and miR-107 MREs within the human S-endoglin mRNA. Therefore, we hypothesize that some or all of the miR-15/107 family members may regulate endoglin expression. Again, it is not clear why the Diana-microT-CDS and TargetScan algorithms did not identify the miR-20a-3p, miR-23b-5p, miR-29a-5p, miR-103-3p, miR-107 and miR-532-5p MREs in human S-endoglin mRNA that were detected manually.

The IMPACT-Seq technique was utilized to experimentally demonstrate that miR-522-3p can regulate human endoglin expression and that the MRE for this miRNA was localized to the 5′-UTR region of human endoglin mRNA isoforms (Tan et al., 2014[153]) (Table 7(Tab. 7)). These investigators found that miR-522-3p typically interacts with noncanonical MRE sequences which contain motifs partially complementary to both the 5′ and 3′ ends of this miRNA. Therefore, the human S-endoglin mRNA was manually screened for the miR-522-3p MRE. The proposed interaction site (5′ CUUCUCUAAGGAAGCGCAUUUC 3′, the partially complementary motifs are underlined) was identified 40 nts downstream from the transcription initiation start site. Given that this predicted MRE is harbored in the 5′-UTR region of the human endoglin mRNA isoforms, miR-522-3p/endoglin mRNA interactions would not be identified by the target algorithms discussed above since they are not programmed to analyze this region. Furthermore, with the tendency of miR-522-3p to interact with noncanonical MRE sequences, Tan et al. (2014[153]) demonstrated that of the 2,467 3′-UTR miR-522-3p MREs that they identified only 111 were predicted by target algorithms. 

In conclusion, it is important to note that although only two of the ten DIANA-TarBase v7.0 cataloged experimentally identified miRNA/human endoglin mRNA interactions (Table 7(Tab. 7)) (Selbach et al., 2008[145]; Balakrishnan et al., 2014[14]; Haecker et al., 2012[63]; Tan et al., 2014[153]) were predicted by miRNA target algorithms, manual sequence inspection detected potential binding sites for all of these miRNAs. Given that the functional importance of the putative MRE(s) for each miRNA described above has not been verified, the biological relevance of each site can be experimentally validated by fulfilling four well-defined experimental criteria defined below. Since the miRNA/human endoglin mRNA interactions have already been experimentally supported (Table 7(Tab. 7)), there is less concern for wasted time and resources testing “false positive” miRNA/mRNA predicted targets (Vlachos and Hatzigeorgiou, 2013[163]).

## Analysis of the Published Literature: the Endoglin Example Revisited

For this review article we subjected the human endoglin mRNA to Diana-microT-CDS and TargetScan miRNA target analyses and examined DIANA-TarBase v7.0 data sets of cataloged and published experimentally supported miRNA/human endoglin mRNA interactions (see above). Given that the majority of the identification and cataloging of miRNA/mRNA target interactions by DIANA-TarBase v7.0 result from high-throughput techniques (Vlachos et al., 2015[164]) without further functional analyses, it is important to examine the published literature to determine that a mRNA of interest is regulated by identified miRNAs (low-yield techniques). In addition, literature may reveal whether miRNAs identified by target algorithm searches and DIANA-TarBase v7.0 have been demonstrated to regulate other mRNA targets (Figure 2(Fig. 2)). Manually curating this data will serve as an important additional step in allowing the investigator to develop the most biologically compelling “experimental hypotheses”.

In our test case, the key words “endoglin” and “miRNAs” were evaluated by PubMed (http://www.ncbi.nlm.nih.gov/pubmed). Six publications were identified (Table 8(Tab. 8); References in Table 8: miR-5739: Yoo et al., 2011[173]; miR-6087: Yoo et al., 2012[174]; miR-208a-5p: Shyu et al., 2013[147]; miR-208a-5p: Wang et al., 2014[165]; miR-15 family: Tijsen et al., 2014[155]; miR-370-3p: Chen et al., 2014[30]). None of these publications were curated by DIANA-TarBase v7.0. Yoo et al., (2011[173], 2012[174]) cloned and characterized two novel miRNAs from human embryonic stem cells that were designated miR-5739 and miR-6087. These investigators demonstrated that the human S-endoglin mRNA isoform harbored a functional miR-5739 and miR-6087 3′-UTR MRE (5′ GCUCUCCG 3′ and 5′ CCCGCCUC 3′, 8mer “seed” regions) located 348 and 366 nts downstream from the stop codon of this mRNA isoform (Yoo et al., 2011[173], 2012[174]). Although both miRNAs have been annotated (miRBase Release 21, accession #: MI0019412 and MI0020364), DIANA-microT-CDS and TargetScan did not predict miR-5739/human endoglin and miR-6087/human endoglin mRNA target interactions since these miRNAs have yet to be included in these algorithms (Garcia et al., 2011[57]; Paraskevopoulou et al., 2013[128]; Reczko et al., 2012[136]).

Shyu et al. (2013[147]) demonstrated that mechanical stretch and TGFβ1 increased miR-208a-5p and endoglin mRNA and protein expression in rat cardiac myoblasts (Table 8(Tab. 8)). This same laboratory also established that miR-208a-5p and endoglin expression was up-regulated in an *in vivo *volume overload-induced heart failure rat model (Wang et al., 2014[165]) (Table 8(Tab. 8)). Importantly, several recent studies have shown that, in addition to targeting mRNAs for translational repression and/or destabilization by the miRISC, miRNAs may also function to induce gene expression by direct interactions with MRE sequences harbored within active promoters or by triplex structure formation (double-stranded DNA/RNA) stabilized by AGO2 (Dharap et al., 2013[38]; Ma et al., 2010[105]; Majid et al., 2010[107]; Place et al., 2008[133]; Toscano-Garibay et al., 2014[156]; Zhang et al., 2014[180]). Shyu et al. (2013[147]) speculate that miR-208a-5p may interact with a MRE located in the promoter region of the rat Eng gene, which in turn induces rat endoglin gene expression. Unfortunately, the predicted rat Eng promoter MRE does not show significant complementarity to miR-208a-5p and does not follow seed sequence rules. These authors have yet to test the biological activity of this site (Shyu et al., 2013[147]). Therefore, it is possible that the elevated endoglin levels could be the result of secondary regulatory events (i.e. down-regulation of a miR208a target that results in elevated endoglin expression). Additionally, mouse endoglin mRNAs harbor a miR-208a-5p MRE located within the 3′-UTR that was predicted by Diana-microT-CDS (0.517 miTG score). This site is conserved in rats but not in humans. Given that translational up-regulation by miRNAs has also been observed as a result of the direct action of miRNAs (reviewed in Valinezhad Orang et al., 2014[158]; Vasudevan, 2012[160]), it is possible that miR-208a-5p binding to this predicted MRE results in the detected up-regulation of rat endoglin. Taken together it is clear that more studies are needed to determine the mechanism by which miR-208a-5p regulates rat endoglin gene expression and to investigate whether this mechanism is also employed in regulating human ENG gene expression.

miRNA profiling expression experiments utilizing ovarian cancer cells and ovarian cancer clinical samples demonstrated that a number of miRNAs were aberrantly expressed, including miR-370-3p, which was down-regulated in these studies (Iorio et al., 2007[76]; Lee et al., 2012[91]). Given that endoglin is known to be over-expressed in some cancers (Rosen et al., 2014[139]), Chen et al. (2014[30]) analyzed the human endoglin mRNA for the presence of miR-370-3p MRE(s) sequences by the TargetScan algorithm (Table 8(Tab. 8)). A miR-370-3p 3′-UTR MRE (5′ CCAGCAGG 3′, 8mer “seed” region, -0.21 total context score) was predicted 256 nts downstream from the stop codon of the human S-endoglin mRNA isoform (Chen et al., 2014[30]). After identifying this MRE, Chen et al. (2014[30]) subsequently demonstrated that human endoglin was negatively regulated by miR-370-3p directly interacting with this sequence (Table 8(Tab. 8)). We also surveyed the 259 Diana-microT-CDS predicted human S-endoglin MRE sequences and found that this algorithm also predicted the same miR-370-3p 3′-UTR MRE (0.519 miTG score) interaction site. However, this algorithm identified one additional miR-370-3p 3′-UTR MRE (5′ CCCCAGCAAGC 3′, 8mer “seed” + mismatch region, underlined) and the potential functionality of this site has not been tested.

Many research studies have revealed that miRNAs are important regulators of cardiac development and play essential roles in cardiovascular diseases (reviewed in Small et al., 2010[149]). Importantly, miRNA expression profiling experiments have identified a subset of miRNAs expressed in the normal heart and which are modulated during cardiovascular disease, including the miR-15/107 family described above (Hullinger et al., 2012[73]; Nigam et al., 2010[123]; Porrello et al., 2011[134], 2013[135]; van Rooij et al., 2006[159]; Zampetaki et al., 2014[177]). 

Tijsen et al. (2014[155]) (Table 8(Tab. 8)) focused their attention on the miR-15/107 family since some members are expressed in both cardiomyocytes and fibroblasts (Hullinger et al., 2012[73]). Mouse TargetScan analysis predicted miR-15/107 family 3′-UTR MREs in canonical TGFβ (TGFβR1, TGFβR2, TGFβR3, endoglin, SMAD2, SMAD3, SMAD4, SMAD7), and in non-canonical TGFβ (TGFβR1, TGFβR2, TRAF6, TAK1, p38) signaling pathways (Tijsen et al., 2014[155]). Notably, mouse endoglin mRNA was predicted to harbor a miR-15/107 family 3′-UTR MRE (5′ UGCUGCU 3′, 7mer “seed” region, -0.18 total context score) located 442 nts downstream from the stop codon. Luciferase reporter assays suggest direct targeting of these miRNAs within the mouse endoglin 3′-UTR (Tijsen et al., 2014[155]) (Table 8(Tab. 8)). Tijsen et al. (2014[155]) also demonstrated that when mice were injected subcutaneously with locked nucleic acid (LNA)-based antimiR-15b, the loss of the miR-15 family members (miR-15-5p, miR-16-5p, miR-195-5p, miR-322 (mouse homolog to human miR-424-5p), and miR-497-5p resulted in a significant up-regulation of TGFβR1 and SMAD3 mRNA, and a trend towards up-regulation of p38, TGFβR2, TGFβR3, SMAD4, SMAD7, and endoglin mRNA. Additionally, when rat neonatal cardiomyocytes were transfected with miR-15b mimics, the overexpression of this miRNA resulted in decreased mRNA levels of p38, SMAD2, SMAD3, and endoglin (Tijsen et al., 2014[155]). Taken together, these investigators concluded that the miR-15/107 family is a novel regulator of cardiac hypertrophy and fibrosis through the inhibition of the TGFβ-signaling pathway (Tijsen et al., 2014[155]). 

Again, it is important to note that the miR-15/107 family members, miR-16-5p, miR-103a-3p, and miR-107 were identified to interact with human endoglin mRNAs by the HITS-CLIP technique (Table 7(Tab. 7)) (Balakrishnan et al., 2014[14]). Additionally, the Diana-microT-CDS algorithm predicted that human S-endoglin mRNAs harbor three miR-16-5p MREs, one 3′-UTR and two CDS interaction sites (Table 7(Tab. 7), and see discussion above). Interestingly, Tijsen et al. (2014[155]) only utilized TargetScan to interrogate the human, mouse, and rat endoglin mRNAs and came to the conclusion that only mouse endoglin mRNAs harbor a miR-15/107 family MRE. Therefore, their data regarding the decreased endoglin mRNA expression in miR-15b mimic transfected rat neonatal cardiomyocytes (Tijsen et al., 2014[155]) is confusing given that the miR-15/107 family 3′-UTR MRE is not conserved in rat endoglin mRNA. This observation prompted us to investigate whether the Diana-microT-CDS algorithm would identify putative miR-15/107 family 3′-UTR MREs harbored in the mouse and/or rat endoglin mRNAs. This algorithm predicted the same MRE sequence (0.510 miTG score) within mouse endoglin mRNA as TargetScan. However, Diana-microT-CDS found that this site was conserved in rat endoglin mRNAs and actually predicted an additional miR-15/107 family 3′-UTR MRE within mouse endoglin mRNAs (5′ UGCUGCU 3′, 7mer “seed” region) located 864 nts downstream from the stop codon.

Although Tijsen et al. (2014[155]) demonstrated that multiple miR-15/107 family members, including miR-16-5p, were up-regulated in human diseased heart samples they did not investigate whether or not endoglin mRNA and/or protein levels were reduced in these samples, especially since their TargetScan analyses suggested that human endoglin would not be regulated by this miRNA family. This was an important oversight given that, like mouse and rat endoglin mRNAs, human endoglin mRNA isoforms harbor algorithm-identified miR-15/107 family MREs and therefore may also be regulated by miR-15/107 family members. Taken together, our endoglin case study clearly demonstrates the importance of utilizing multiple target algorithms and the data curated by DIANA-TarBase v7.0, in conjunction with the published literature in order to appropriately interpret miRNA data.

Given that endoglin has been established to play a regulatory role in TGFβ signaling (reviewed in Kapur et al., 2013[78]; López-Novoa and Bernabeu, 2010[102]; Nachtigal et al., 2012[119]; Rosen et al., 2014[139]), and since aberrant TGFβ signaling can play a role angiogenesis, atherosclerosis, atrial fibrillation, cancer, diabetes mellitus, heart failure, hereditary hemorrhagic telangiectasia (HHT), myocardial infarction, pre-eclampsia, and pulmonary hypertension (Kapur et al., 2013[78]; López-Novoa and Bernabeu, 2010[102]; Nachtigal et al., 2012[119]; Rosen et al., 2014[139]), it follows that one should also examine the published literature to investigate whether or not any of the algorithm computed miRNAs (259 Diana-microT-CDS [Table 1(Tab. 1)], 152 TargetScan miRNA/miRNA families [Table 2(Tab. 2)]) and the experimentally cataloged miRNAs (Table 7(Tab. 7)) predicted to interact with human endgolin mRNAs, have been shown to play a role in any of the pathologies described above. Therefore, each identified miRNA was utilized as a key word and interrogated by PubMed. miRNA searches that resulted in over forty “hits” were re-analyzed utilizing the given miRNA and the listed pathologies described above. 

Importantly, members of the miR-15/107 family have been demonstrated to play key roles in gene regulation involved in cell division, metabolism, stress response, and angiogenesis (reviewed in Finnerty et al., 2010[53]). This family has also been implicated in pathological processes including cancers, cardiovascular disease and neurodegenerative diseases (Finnerty et al., 2010[53]). Additional miRNAs with potential roles in regulating endoglin biology include miR-628-5p (Table 6(Tab. 6)) given that it is down-regulated in prostate cancer (Srivastava et al., 2014[150]) and the miR-15/107 family and miR-628-5p that are regulated by IL-3, GM-CSF and G-CSF in acute myeloid leukemia (Favreau et al., 2012[52]). Further, let-7b-5p, miR-20a, and miR-29a-5p (experimentally cataloged miRNAs which target human endoglin mRNAs, Table 7(Tab. 7)) are potent tumor suppressors which are involved in cell proliferation, cell cycle regulation, and have been associated with increased tumor metastasis (Fabbri et al., 2007[47]; Pickering et al., 2009[132]; Yun et al., 2011[176]). In contrast, miR-23b-5p, miR-522-3p, and miR-532-5p (experimentally cataloged miRNAs which target human endoglin mRNAs, Table 7(Tab. 7)) appear to have metastatic-promoting functions (Ell et al., 2014[46]; Kitago et al., 2009[85]; Tan et al., 2014[152]). Finally, several human endoglin mRNA algorithm computed interacting miRNAs that might be interesting to investigate include miR-26a/b-5p, miR-93-5p, miR-150-5p, miR-326, miR-370 given that these miRNAs have been shown to have tumor suppressor/promoter and cardiovascular roles (Chen et al., 2014[30]; Fang et al., 2011[50]; Icli et al., 2014[74]; Ito et al., 2014[77]; Kim et al., 2014[84]; Lo et al., 2012[100]; Lyu et al., 2014[104]; Zeitels et al., 2014[178]).

## Develop Experimental Hypotheses: the Continued Endoglin Case Study

Once the plethora of information from prediction algorithms, published validations of miRNA/mRNA interactions, and manual sequence inspections of miRNA binding sites has been assembled, prioritization of specific miRNA/mRNA target interactions to investigate can more effectively proceed. Among the number of putative miRNA/human endoglin mRNA interactions documented above, the remainder of this review article will focus on miR-370 (Table 8(Tab. 8)) since this miRNA has already been identified and validated to interact with endoglin mRNA and negatively regulate endoglin protein expression (Chen et al., 2014[30]). We will utilize this published example to outline and explain the four criteria/experimental procedures to thoroughly validate a miRNA/ mRNA target interaction as biologically significant. 

## Validation of miRNA/mRNA Interactions

### Demonstrate miRNA and target mRNA co-expression in vivo

Clearly a given miRNA and its target mRNA must be co-expressed in order for the miRNA to regulate the expression of a given biological target. Therefore, miRNA and target mRNA co-expression experimental studies should be performed first (Figure 2(Fig. 2)), since there is no need to proceed with additional validation experiments if a tissue or cell type can't be identified where they are co-expressed. 

Co-expression is typically demonstrated by simply performing Northern blot analysis or quantitative real-time PCR (qPCR) using total RNA isolated from a specific cell type or tissue, and probes or primers specific for a given miRNA and mRNA target (Sansom et al., 2011[142]). We recommend that qPCR experiments be performed given that many commercial assays are available to measure both miRNA (Life Technologies, Exiqon, Qiagen) and mRNA (Life Technologies, Qiagen) levels from many species and due to the ease and reproducibility of these assays.

If expression data regarding the miRNA and/or target mRNA of interest is scarce, then many tissues and/or cell lines may need to be screened (Sansom et al., 2010[142]). Additionally, if cell specific expression information concerning the miRNA and/or target mRNA of interest is unknown, then it may also beneficial to perform miRNA and mRNA *in situ* hybridization and immunohistochemical experiments utilizing paraffin-embedded, formalin-fixed tissues to address the question of co-expression (Nuovo, 2010[124]; Sansom et al., 2010[142]).

As described in the “Analysis of the Published Literature” section above (Table 8(Tab. 8)), recent reports suggest that miR-370 is a tumor suppressor (An et al., 2012[6]; Iorio et al., 2007[76]; Lee et al., 2012[91]; Zhang et al., 2012[179]). Given that angiogenesis is required for the survival and growth of solid cancers (reviewed in Carmeliet, 2003[29]) and since endoglin is essential for angiogenesis (Dallas et al., 2008[34]), Chen et al. (2014[30]) investigated whether human endoglin mRNAs harbored putative miR-370 MREs by utilizing the TargetScan algorithm. A miR-370-3p 3′-UTR MRE (5′ CCAGCAGG 3′, 8mer “seed” region) was predicted 256 nts downstream from the stop codon in the human S-endoglin mRNA isoform (Chen et al., 2014[30]). It is important to note that of the 249 miRNA MRE sites predicted by TargetScan in the human S-endoglin mRNA isoform, miR-370-3p had only the 40th highest total context score (-0.21). Thus, the decision by these investigators to test the hypothesis that miR-370-3p can regulate endoglin expression was based on the published observations that this miRNA target might be biologically relevant even though the context score for miR-370-3p was not that striking (Table 2(Tab. 2)).

Importantly, Chen et al. (2014[30]) initiated their study by investigating whether or not human ovarian cancer tissues and endometrioid ovarian cancer cell lines expressed endoglin and miR-370-3p. Northern blot and qPCR experiments demonstrated that, compared with normal ovarian tissues and control ovarian epithelial cells, miR-370-3p expression levels were attenuated in endometrial ovarian cancer tissues and in two endometrioid ovarian cancer cell lines (IGROV1 and TOV112D) (Chen et al., 2014[30]). Additionally, these investigators utilized immunohistochemical and Western blot experiments to demonstrate that endoglin was expressed in normal ovarian tissue and in IGROV1 and TOV112D cells (Chen et al., 2014[30]). Taken together, these data indicated that miR-370-3p and endoglin were co-expressed in ovarian tissues and cells. Further evaluation of the biological importance of the miR-370-3p MRE harbored in the human S-endoglin mRNA isoform is warranted.

### Demonstrate interaction of miRNA to a specific MRE target site

After the demonstration of co-expression of the miRNA and target mRNA of interest, the physical interaction of a specific miRNA with a candidate MRE harbored in a target mRNA needs to be confirmed (Figure 2(Fig. 2)). The majority of MRE validation studies employ co-transfection experiments using chimeric luciferase reporter plasmid constructs which harbor a wild-type or mutated MRE of interest and reagents that either up-regulate (i.e., gain-of-function) or inhibit miRNA activity (i.e., loss-of-function) to rapidly, reliably, and quantitatively screen MRE target-sites (Nicolas et al., 2011[121]; Sansom et al., 2010[142]). The rationale for performing these types of experiments is based on the principle that if a given mRNA is a true target of a specific miRNA, then manipulation of endogenous miRNA concentrations should correspond to predictable changes in target protein levels (i.e. luciferase reporter levels/activity) (Sansom et al., 2010[142]). 

For construction of chimeric luciferase reporter constructs, the predicted MRE sequence from the target gene, most often located in the 3'-UTR but also in the 5'-UTR and CDS see (Akhtar et al., 2015[3]; Zhou and Rigoutsos, 2014[181]), is cloned immediately downstream of the luciferase (Photinus or Renilla) open reading frame sequence contained in the reporter plasmid (Nicolas et al., 2011[121]; Sansom et al., 2010[142]). If possible, it is important that the entire 3′-UTR be included since a truncated version of this sequence may provide inappropriate accessibility to a given miRNA. Additionally, by sub-cloning the entire 3′-UTR of the target gene of interest, a single reporter construct can be utilized to investigate most of the algorithm-predicted miRNA/mRNA binding sites. For many human and rodent target mRNAs, chimeric luciferase reporter constructs containing the entire 3′-UTR can be obtained from several commercial sources (e.g., GeneCopoeia, Inc; OriGene Technologies, Inc; SwitchGear Genomics). We recommend the utilization of a dual-reporter vector system (e.g., psiCHECK-2, Promega; pEZX-MT05, pEZX-MT06, GeneCopoeia, Inc) since this enables transfection normalization for accurate across-sample comparison by transfecting a single plasmid (Sansom et al., 2010[142]). 

Once the wild-type and mutant MRE chimeric luciferase reporter constructs have been generated, consideration must be given to augmentation or attenuation of the cellular levels of a given mature miRNA. Briefly, miRNA mimics, which are utilized for gain-of-function experiments, are chemically synthesized as double-stranded RNA oligonucleotides which simulate the Dicer cleavage product (Figure 1(Fig. 1)) after transfection into cells. These transfected mimics are subsequently processed into mature miRNAs (guide strand) such that the passenger strand (antisense to the guide strand) is excluded through a proprietary chemical modification pattern (commercially available from Ambion/Life Technologies, Dharmacon, Qiagen). Alternatively, miRNA mimics can be synthesized in three strands (Bramsen et al, 2007[24]) rather than the two strand mimics described above. These mimics are comprised of a mature miRNA (guide strand) that is a chemically synthesized unmodified RNA oligonucleotide strand with a sequence corresponding exactly to the annotation in miRBase (http://www.mirbase.org) and a passenger strand that is split in two separate antisense chemically synthesized LNA modified RNA oligonucleotide strands (commercially available from Exiqon). After transfection into cells, the segmented nature of the passenger strand ensures that only the mature miRNA (guide strand) is loaded into the RISC with no resulting miRNA activity from the passenger strand. Regardless of the chemical makeup of the mimic utilized, transfection of a miRNA mimic into cells will increase the proportion of RISC containing this particular miRNA and therefore, gain-of-function studies can assess the biological consequences (i.e. repression of luciferase reporter levels/activity) resulting from an increase in the activity of the mimicked miRNA (Sansom et al., 2010[142]). 

In contrast, miRNA inhibitors, which are utilized for loss-of-function experiments, are chemically synthesized, single-stranded, modified antisense RNA oligonucleotides which are designed to bind with and form highly stable heteroduplexes with the complementary endogenous miRNAs when introduced into cells (Meister et al., 2004[113]; Sansom et al., 2010[142]). As a consequence, the formation of heteroduplexes effectively prevents this miRNA from hybridizing with its normal cellular mRNA targets. Therefore, loss-of-function studies can assess the biological consequences (i.e. de-repression of luciferase reporter levels/activity) due to a decrease in the activity of a selected miRNA (Sansom et al., 2010[142]). 

Despite widespread use of chimeric luciferase reporter genes, miRNA gain-of-function experiments can result in misleading assessment of targets since transient transfection of miRNA mimics generally results in supra-physiological concentrations of miRNAs that may potentially generate false positive results (Arvey et al., 2010[9]; Bracken et al., 2008[23]). For example, exaggerated miRNA over-expression can potentially saturate miRISC complexes and displace other endogenous miRNAs and consequently cause low afﬁnity target sites to appear functionally important (Khan et al., 2009[82]). Therefore, it is important that miRNA gain-of-function transfection experiments be optimized to deliver the minimal required mRNA mimic concentration for validation of a biological effect. Additionally, the appropriate negative and positive control experiments need to be performed to ensure that the resulting change in luciferase activity is due to the increased activity of the mimicked miRNA. For example, negative control chimeric luciferase reporter transfection experiments should always be performed utilizing scrambled miRNA mimics to demonstrate the specificity of a given miRNA for a MRE localized in the mRNA target of interest (Sansom et al., 2010[142]). Finally, miRNA over-expression experiments are often performed in a cell environment that is artiﬁcial to the chosen miRNA. However, due to tissue-specific miRNA biogenesis and binding (Kedde et al., 2007[80]; Siomi and Siomi, 2010[148]), these studies should be ideally performed in relevant cell lines that express the investigated miRNA and the mRNA of interest.

For the endoglin case study, to investigate miR-370-3p interaction with the predicted MRE site harbored in the 3′-UTR of human endoglin mRNA, Chen et al. (2014[30]) engineered two chimeric luciferase/endoglin reporter gene constructs. The wild-type chimeric construct harbored a small portion of the human endoglin 3′-UTR (29 nts) including the miR-370-3p MRE and an identical chimeric construct in which the miR-370-3p MRE seed sequence was mutated. The two human endometrioid ovarian cancer cell lines described above were individually transfected with these reporter constructs and miR-370-3p mimics and luciferase activity was measured. Cells transfected with the wild-type chimeric luciferase/endoglin reporter construct and miR-370-3p mimic exhibited the lowest luciferase activity (Chen et al., 2014[30]). In contrast, the luciferase activity was unchanged in cells transfected with the mutant chimeric luciferase/endoglin reporter construct and miR-370-3p mimics. Since the transfected ovarian cancer cells endogenously express miR-370-3p, these investigators also performed identical luciferase reporter transfection experiments utilizing a miR-370-3p inhibitor. Notably, these transfection experiments demonstrated that luciferase reporter activity was increased in cells transfected with the wild-type chimeric luciferase/endoglin reporter construct and the miR-370-3p inhibitor (Chen et al., 2014[30]). Taken together, these results indicate that miR-370-3p can mediate luciferase mRNA translational repression and/or destabilization by directly interacting with the predicted miR-370-3p MRE target site. 

### Demonstrate miRNA mediated effects on target protein expression

Although the ability of miRNAs to repress the activity of a chimeric luciferase reporter gene is a useful screening device, it remains a surrogate assay for testing the effects of miRNAs on their putative mRNA targets. Therefore, after confirming the physical interaction of a miRNA with a candidate MRE harbored in target mRNAs by reporter assays, we recommend that miRNA gain- and loss-of-function transfection experiments also be performed to validate miRNA-mediated post-transcriptional regulation of target genes of interest (Figure 2(Fig. 2)). 

Experimental manipulation of endogenous miRNA activity by miRNA mimic and miRNA inhibitor transfection should correspond to predictable changes in target protein levels (normally by Western blotting, ELISA, immunostaining, etc.) (Sansom et al., 2010[142]). Therefore, when a given mRNA is an authentic miRNA target, transfection of that miRNA mimic into a cell type known to express the putative target protein, should result in decreased target protein expression due to increased mature miRNA activity (i.e., gain-of-function) (Sansom et al., 2010[142]). In contrast, transfection of a specific miRNA inhibitor into a cell type known to co-express the target protein and miRNA of interest, should result in augmented target protein expression as a result of decreased endogenous miRNA activity (i.e., loss-of-function) (Sansom et al., 2010[142]). It is important to note that loss-of-function experiments can also be achieved by utilizing plasmid constructs which express mRNAs containing multiple artificial miRNA-binding sites, which act as decoys or “sponges” (Ebert and Sharp, 2010)[44]. Over-expression of the mRNA-specific sponges selectively sequesters endogenous miRNAs and as a consequence target protein levels increase (Tay et al., 2015[154]). 

Although gain- and loss-of-function experiments are powerful, it is important to remember that results can be confounded by side effects of transfection (Arvey et al., 2010[9]; Bracken et al., 2008[23]; Khan et al., 2009[82]) and secondary effects resulting from the change in miRNA activity (Matkovich et al., 2013[111]; Riba et al., 2014[138]). For example, miRNAs may directly or indirectly affect the activity of multiple transcription factors which in turn can have profound effects on transcription that are not the direct result of miRNA interaction with mRNA targets (Matkovich et al., 2013[111]; Riba et al., 2014[138]). The advantage of miRNA inhibitor loss-of-function experiments is that they will reveal whether or not the observed direct interaction of a given miRNA to a specific MRE target site, based on the luciferase reporter assays, is relevant in the studied biological context; not exclusively significant when the miRNA is artificially expressed at high levels (Meister et al., 2004[113]; Sansom et al., 2010[142]).

The choice of cells utilized for gain- and loss-of-function studies is critical as each cell line has varying levels of endogenous miRNA and target gene expression. It is important to select a cell culture system that expresses an appropriate level of endogenous miRNA and target gene so that the effects of the miRNA mimics and inhibitors on protein levels can be clearly detected. For example, since miRNA mimics repress target gene protein expression, mimics produce the best results in cells that express low levels of endogenous miRNAs and correspondingly high target mRNA expression (Sansom et al., 2010[142]). Under these conditions, when a given miRNA mimic is over-expressed, repression of the target protein should be easily detectable. In contrast, if experiments are performed in cells with high endogenous miRNA levels and correspondingly low target expression, the effects of miRNA mimic over-expression on the target gene may not be detectable. Instead, these cells are best for investigating the effects of miRNA inhibitors since the resulting de-repression of target protein expression will be more pronounced and easily quantified in these cells. 

While gain- and loss-of-function experiments can be used validate miRNA/mRNA target interactions, it is also possible to utilize the IMPACT-Seq experimental methodology described in the “Analysis of experimentally validated miRNA/mRNA target data bases” section above, to independently validate whether or not a given miRNA can bind to specific mRNAs *in vivo* (Tan et al., 2014[153]). Recall that this miRNA ''pull-down'' strategy introduces specific biotinylated miRNA mimics into cells and miRNA/mRNA targets are pulled down utilizing streptavidin (Tan et al., 2014[153]). These products are treated with RNase and the RNase-resistant fragments are subjected to NGS to identify the pulled-down miRNA/mRNA targets and to characterize specific MRE(s) located within the targeted mRNAs (Tan et al., 2014[153]). Alternatively, once the miRNA/mRNA targets have been pulled down by streptavidin, specific mRNAs can be identified by qPCR utilizing primers for the gene target of interest (Subramanian et al., 2015[151]). Therefore, this approach provides a means to identify functional miRNA targets based on their physical interaction *in vivo*. Since ''predetermined'' target genes are being characterized, this procedure could be utilized to validate whether or not a given mRNA of interest is interacting with a specific miRNA *in vivo*. Additionally, these experiments can be used to validate the functional MREs identified by chimeric luciferase reporter assays described above. Furthermore, miRNA ''pull-down'' assays can also be utilized to identify MREs harbored in the 5′-UTR and CDS of target mRNAs. Finally, given that recent studies have demonstrated that non-canonical miRNA interactions are more prevalent than previously appreciated, miRNA ''pull-down'' assays may provide a novel way to identify MREs that are not predicted by miRNA/mRNA target algorithms (Grosswendt et al., 2014[60]; Helwak et al., 2013[68]; Martin et al., 2014[110]; Tan et al., 2014[153]). 

Chen et al., (2014[30]) demonstrated that miR-370-3p could repress the activity of a chimeric luciferase/endoglin reporter gene. These investigators subsequently performed gain- and loss-of-function experiments to determine whether manipulation of endogenous miR-370-3p activity corresponded to predictable changes in endoglin protein expression. Since the two human endometrioid ovarian cancer cell lines (IGROV1 and TOV112D), expressed both miR-370-3p and endoglin at easily detectable levels, they were able to perform gain- and loss-of-function experiments in each cell line (Chen et al., 2014[30]). In miR-370-3p mimic transfected cells human endoglin protein levels were repressed. In contrast, human endoglin protein levels were augmented in IGROV1 and TOV112D cells transfected with miR-370-3p inhibitors. Taken together, these results suggest that the miR-370-3p can bind to the predicted miR-370-3p MRE sequence harbored in the 3′-UTR of human endoglin mRNA and imply that this interaction is physiologically relevant. “Pull-down” experiments were not performed that may have proven useful since the Diana-microT-CDS algorithm predicted that human S-endoglin mRNA harbors an additional miR-370-3p 3′-UTR MRE whose function is unknown.

### Demonstrate miRNA effects on biological function

After miRNA gain- and loss-of-function transfection experiments have confirmed that a given miRNA mimic and inhibitor mediate the inverse protein expression of a target gene of interest, it is finally necessary to demonstrate that this regulation equates to changes in biological function (Figure 2(Fig. 2)). Depending upon the protein target of interest, a variety of biological assays could be performed including signaling pathway evaluations, cell proliferation, cell differentiation, cell death, cell migration, receptor binding, etc. (Sansom et al., 2010[142]). Importantly, when a biological pathway is being studied, phenotypic changes may be assayed as an indirect measure of miRNA effects on target protein levels (i.e., gain- and loss-of-function transfection experiments) as long as the phenotypic assay is accompanied by a direct protein assay.

The wide ranging biological effects of a given miRNA can also be investigated by performing *in vivo* gain- and loss-of-function experiments in mice or rats (reviewed in Hinkel et al, 2014[69]; Li and Rana, 2014[99]). For example, miRNA function can be increased by directly infusing specific miRNA mimics (Di Martino et al., 2014[39]; Montgomery et al., 2014[116]), by infusing miRNA mimics packaged in lipid-based nanoparticles (Das et al., 2014[35]; Hsu et al., 2013[71]; Huang et al., 2013[72]), or by the use of adeno-associated viruses (AAV) to drive the forced-expression of a given miRNA (Kasinski and Slack, 2012[79]; Kota et al, 2009[86]; Miyazaki et al, 2012[115]). In contrast, endogenous miRNAs can be silenced by systemic delivery of cholesterol-conjugated miRNA inhibitors, designated “antagomirs” (Martin del Campo et al., 2015[109]; McClure et al., 2014[112]), by infusing LNA miRNA inhibitors (Di Martino et al., 2014[40]; Seeger et al., 2014[144]; Tijsen et al., 2014[155]), or by infusing miRNA inhibitors packaged in lipid-based nanoparticles (Babar et al., 2012[10]; Baigude and Rana, 2012[12]).

Chen et al. (2014[30]) demonstrated that miR-370-3p gain- and loss-of-function experiments inversely regulated endoglin protein expression. They subsequently performed additional gain- and loss-of-function experiments to determine whether or not the experimental manipulation of endogenous miR-370-3p activity corresponded to observable changes in specific biological responses. These experiments demonstrated that miR-370 mimicry suppressed endometrioid ovarian cancer cell malignant phenotypes via the negative regulation of endoglin (Chen et al., 2014[30]). Taken together these investigators hypothesized that, in endometrioid ovarian cancer cells, hypermethylation reduces miR-370 levels which in turn results in the elevated expression of its direct target endoglin. As a consequence, endoglin over-expression contributes to the enhanced malignant properties in endometrioid ovarian cancer cells including high proliferation, low apoptosis and/or cell death, and enhanced chemoresistance.

## Conclusion

miRNAs are emerging as important post-transcriptional regulators of gene expression and consequently are central players in many physiological and pathological processes (e.g., Adams et al., 2014[2]; Iorio and Croce, 2012[75]; Neppl and Wang, 2014[120]; Trionfini et al., 2015[157]). Since the biological roles of miRNAs are dictated by the mRNAs that they regulate, the identification and validation of miRNA/mRNA target interactions is critical for our understanding of the regulatory networks governing biological processes. We advocate the combined use of prediction algorithms, the examination of experimentally supported miRNA/mRNA interactions cataloged from high throughput experimental data sets, manual sequence inspection of cataloged miRNA binding sites in target mRNAs, and a review of the published literature as the optimal practice for identifying and prioritizing the most biologically compelling miRNA/mRNA target pairs based on individual research interests (Figure 2(Fig. 2)). For effectively utilizing these strategies, throughout this review, we have used the example of miRNA regulation of endoglin. 

Once a preferred miRNA/mRNA target pair has been selected, we propose that the authenticity of a functional miRNA/mRNA target pair be validated by fulfilling four criteria. First, the predicted miRNA and mRNA target gene must be co-expressed. Second, direct interaction of a given miRNA to a specific MRE harbored within the target mRNA must be demonstrated. Third, gain- and loss-of-function experiments utilizing miRNA mimics and inhibitors must inversely regulate target protein expression. Fourth, miRNA-mediated regulation of target gene expression (gain- and loss-of-function) should equate to altered biological function. 

To date only a small proportion of miRNA/mRNA target interactions have been functionally validated. The unique experimental outline described here can be applied to the validation of any miRNA/mRNA interaction. As relevant targets are identified, the biological functions of a specific miRNA can be unraveled and assist in development of miRNA therapeutics.

## Conflict of interest

The authors declare that they have no conflict of interest.

## Acknowledgements

We express our appreciation to Ms. Emily Keeler for generating the figures and tables.

## Figures and Tables

**Table 1:**
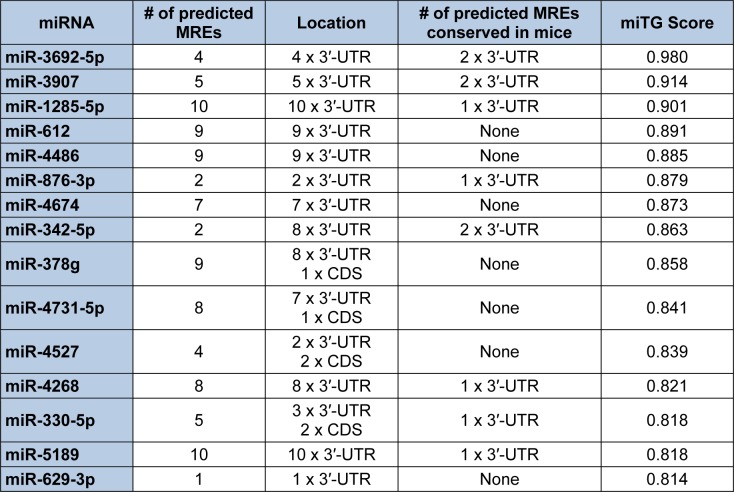
Top fifteen DIANA-microT-CDS predicted human miRNA/endoglin mRNA target interactions based on their targeting score

**Table 2:**
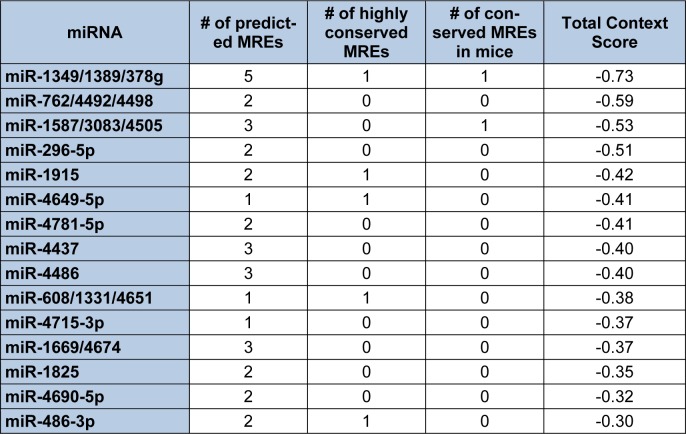
Table 2: Top fifteen TargetScan predicted miRNA/S-endoglin mRNA target interactions based on their target score (Human, ENG, NM 000118, 3′-UTR length 905 nt)

**Table 3:**
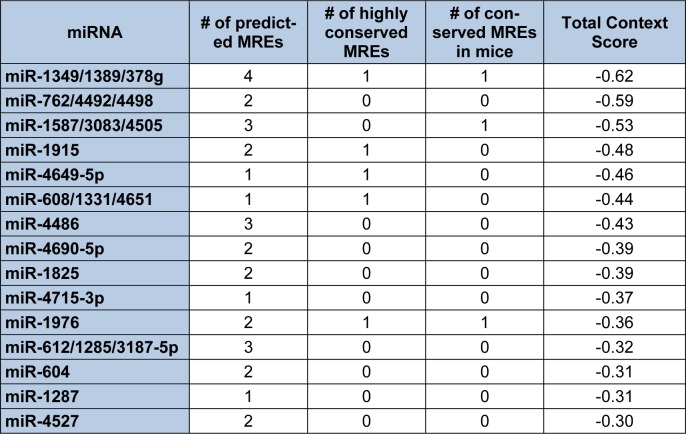
Top fifteen TargetScan predicted miRNA/L-endoglin mRNA target interactions based on total context score (Human ENG, NM_001114573, 3′-UTR length 670 nt)

**Table 4:**
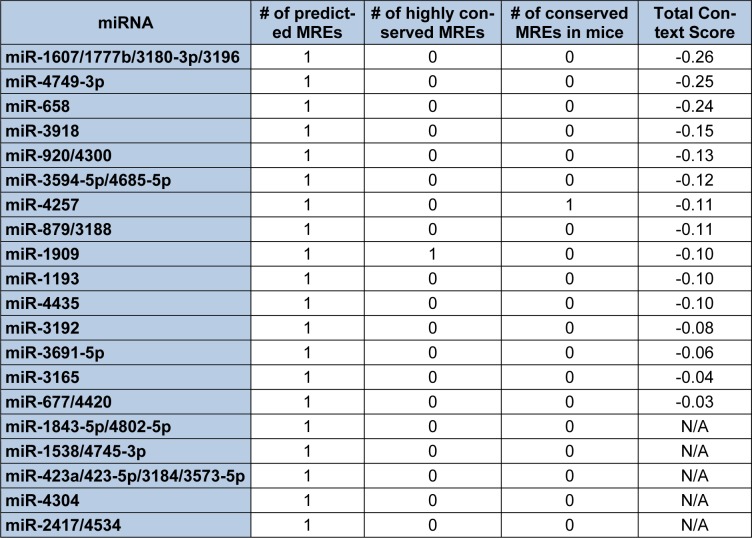
TargetScan identified miRNAs that are predicted to target MREs that are unique to the human S-endoglin mRNA isoform

**Table 5:**
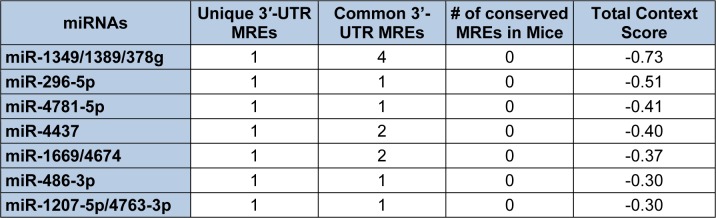
TargetScan identified miRNAs that are predicted to target MREs that interact with the unique and common 3′-UTR regions in human endoglin mRNA isoforms

**Table 6:**
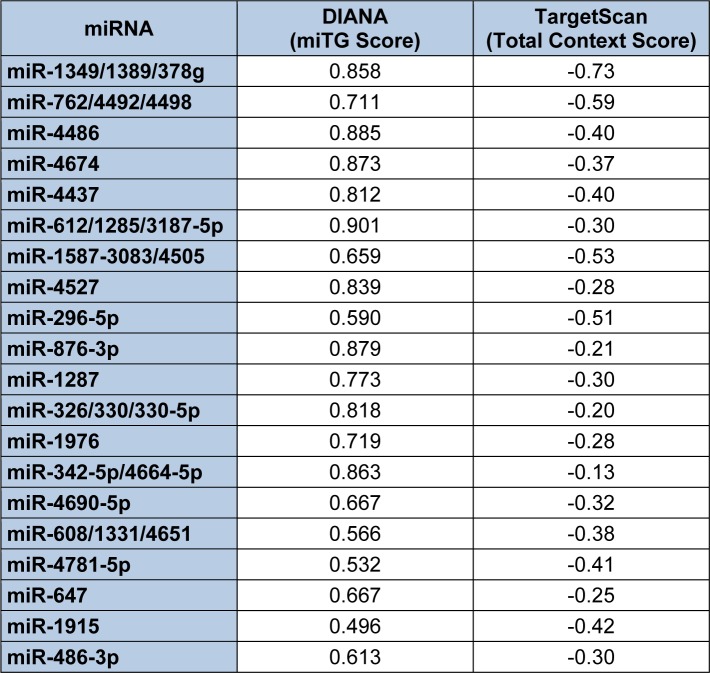
Top 20 miRNA/endoglin mRNA target interactions predicted by both algorithms

**Table 7:**
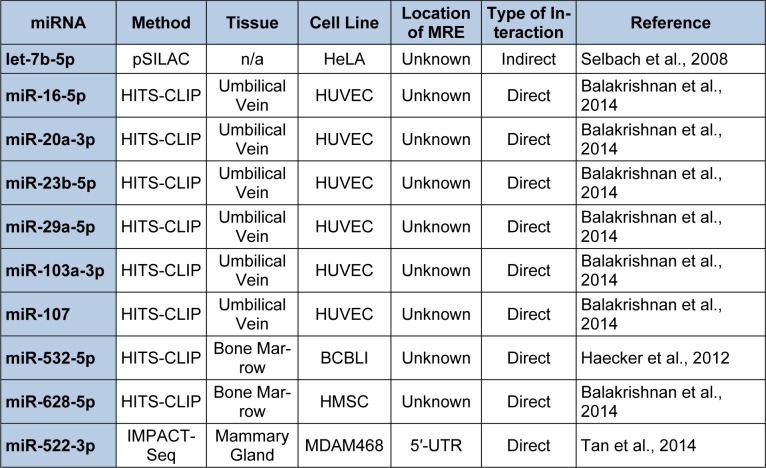
Experimentally supported human miRNA/endoglin mRNA interactions cataloged by DIANA-TarBase

**Table 8:**
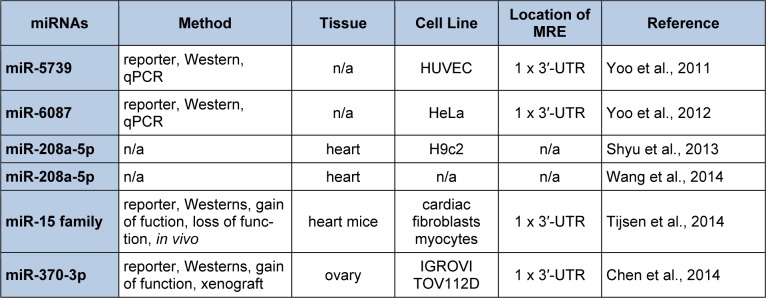
AlI published studies investigating miRNA/endoglin regulation
